# “Balancing Expectations with Actual Realities”: Conversations with Clinicians and Scientists in the First Year of a High-Risk Childhood Cancer Precision Medicine Trial

**DOI:** 10.3390/jpm10010009

**Published:** 2020-02-14

**Authors:** Brittany C. McGill, Claire E. Wakefield, Kate Hetherington, Lachlan J. Munro, Meera Warby, Loretta Lau, Vanessa Tyrrell, David S. Ziegler, Tracey A. O’Brien, Glenn M. Marshall, David Malkin, Jordan R. Hansford, Katherine M. Tucker, Janine Vetsch

**Affiliations:** 1 School of Women’s and Children’s Health, UNSW Medicine, UNSW Sydney, Sydney 2052, Australia; c.wakefield@unsw.edu.au (C.E.W.); k.hetherington@unsw.edu.au (K.H.); lachlan.munro@unsw.edu.au (L.J.M.); loretta.lau@health.nsw.gov.au (L.L.); d.ziegler@unsw.edu.au (D.S.Z.); t.obrien@unsw.edu.au (T.A.O.); j.vetsch@unsw.edu.au (J.V.); 2 Behavioural Sciences Unit, Kids Cancer Centre, Sydney Children’s Hospital, Randwick 2031, Australia; 3 Hereditary Cancer Centre, Department of Medical Oncology, Prince of Wales Hospital, Randwick 2031, Australia; meera.warby@health.nsw.gov.au (M.W.); kathy.tucker@health.nsw.gov.au (K.M.T.); 4 Prince of Wales Clinical School, UNSW Sydney, Sydney 2052, Australia; 5 Kids Cancer Centre, Sydney Children’s Hospital, Randwick 2031, Australia; g.marshall@unsw.edu.au; 6 Children’s Cancer Institute, UNSW Sydney, Kensington 2750, Australia; vtyrrell@ccia.org.au; 7 Division of Haematology/Oncology, Hospital for Sick Children, Department of Paediatrics, University of Toronto, Toronto, ON M5G 1X8, Canada; david.malkin@sickkids.ca; 8 Children’s Cancer Centre, Royal Children’s Hospital, Melbourne 3052, Australia; jordan.hansford@rch.org.au; 9 Division of Cancer, Murdoch Children’s Research Institute, Melbourne 3052, Australia; 10 Department of Paediatrics, University of Melbourne, Melbourne 3010, Australia; 11 Department of Paediatrics, Monash University, Melbourne 3800, Australia

**Keywords:** precision medicine, neoplasms, genomics, pediatrics, delivery of healthcare

## Abstract

Precision medicine is changing cancer care and placing new demands on oncology professionals. Precision medicine trials for high-risk childhood cancer exemplify these complexities. We assessed clinicians’ (*n* = 39) and scientists’ (*n* = 15) experiences in the first year of the PRecISion Medicine for Children with Cancer (PRISM) trial for children and adolescents with high-risk cancers, through an in-depth semi-structured interview. We thematically analysed participants’ responses regarding their professional challenges, and measured oncologists’ knowledge of genetics and confidence with somatic and germline molecular test results. Both groups described positive early experiences with PRISM but were cognisant of managing parents’ expectations. Key challenges for clinicians included understanding and communicating genomic results, balancing biopsy risks, and drug access. Most oncologists rated ‘good’ knowledge of genetics, but a minority were ‘very confident’ in interpreting (25%), explaining (34.4%) and making treatment recommendations (18.8%) based on somatic genetic test results. Challenges for scientists included greater emotional impact of their work and balancing translational outputs with academic productivity. Continued tracking of these challenges across the course of the trial, while assessing the perspectives of a wider range of stakeholders, is critical to drive the ongoing development of a workforce equipped to manage the demands of paediatric precision medicine.

## 1. Introduction

Precision medicine, with its focus on matching recommended treatments to specific pathogenic molecular aberrations, is a promising and paradigm-shifting strategy in childhood cancer treatment [1,2]. Access to precision medicine trials may be particularly impactful for paediatric cancer patients with poor-prognosis malignancies [3,4]. Precision medicine trials can offer hope to families, but are imbued with complexities at both a scientific and healthcare-systems level, introducing new challenges for all stakeholders [2].

Childhood cancer care is emotionally demanding for the healthcare team due to the young age of the patient and the potential for widespread impact on the family, especially if the child dies [5]. Precision medicine has the potential to compound these emotional pressures by demanding new ways of working for non-genetics trained clinicians, including a heightened level of interdisciplinary engagement and increased familiarity with genomic medicine [2,6,7,8,9,10]. Despite growing evidence that clinicians are hopeful about the clinical utility of precision medicine [11], they may not feel adequately skilled to interpret and communicate the results of advanced genomic sequencing to patients and their families [12,13]. Non-genetics-trained clinicians may feel particularly diffident interpreting and utilising information about germline variants in cancer predisposition genes [11,14].

Clinicians face the additional challenging task of managing informed consent consultations and addressing family expectations in a setting that often sits at the junction between research and clinical practice [15,16]. Not surprisingly, there is evidence that patients and their parents may misunderstand the aims of early phase oncology research [17] including the implications of genomic tumour profiling [18,19]. The clinicians’ role in managing families’ expectations is further complicated by evidence that parents may experience psychological benefits from hoping for their child’s survival, despite understanding that direct benefit to their child may be limited [20,21].

In the high-stakes environment of precision medicine for high-risk childhood cancer, scientists aim to determine actionable variants promptly so that the child’s diagnosis and/or treatment plan can be reassessed in a clinically relevant timeframe [1,2]. However, even if a targetable genomic alteration that may be beneficial to the child’s treatment is identified, there are often logistical challenges in accessing targeted drugs due to cost, availability and off-label use [2,22]. If cancer predisposing germline variants are identified, family implications need to be considered [23]. Molecular tumour boards (MTB), which bring together various clinical and scientific specialties to facilitate knowledge exchange and enhance clinical decision-making, have emerged in response to the complexities of precision medicine [7,24]. The discussion of individual patient outcomes in the MTB is likely to be a particularly novel experience for scientists. The potential multidisciplinary communication challenges, professional pressures, and role changes associated with the MTB have seldom been investigated in the literature. 

Despite repeated assertions that stakeholder engagement is critical for the effective implementation of precision medicine into standard healthcare practice [25,26], the experiences of professionals at the frontline of childhood cancer precision medicine trials are understudied. We recently investigated healthcare professionals’ perspectives of a single-site precision medicine pilot program, the aim of which was to assess the feasibility of the program rather than return results to families [22]. The experiences of scientists, who are exposed to new roles and pressures, are particularly under-studied. To bridge this gap, we recruited clinicians and scientists at the commencement of a national precision medicine trial for high-risk paediatric cancer patients to obtain an in-depth understanding of their early experiences and their perceptions of potential professional challenges.

## 2. Materials and Methods

### 2.1. Participants 

#### 2.1.1. The PRISM Trial

**Figure 1:**
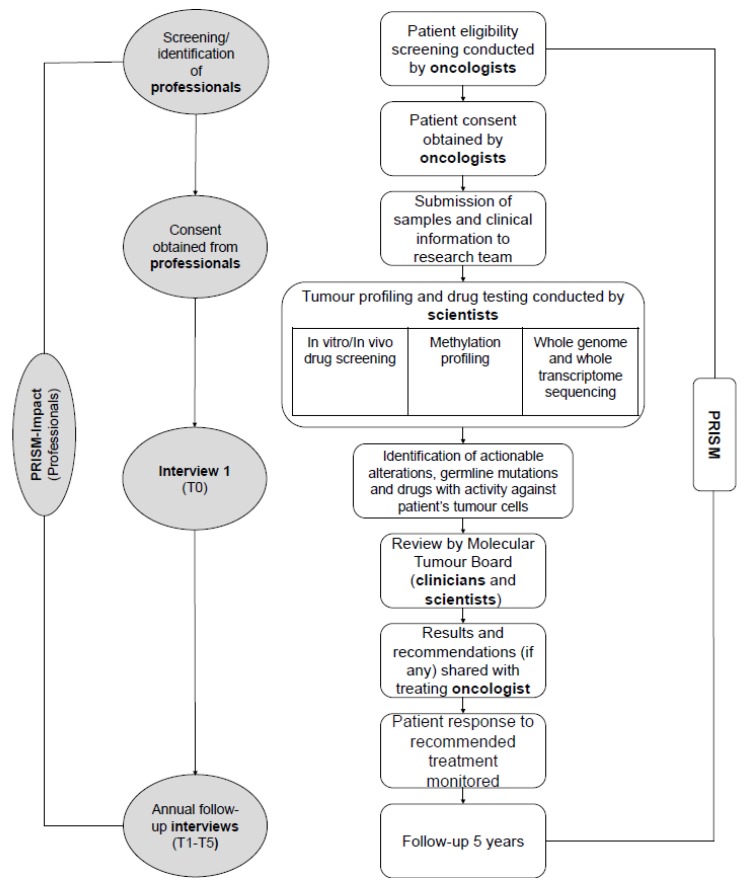
PRISM and PRISM-IMPACT (professionals) study flowchart identifying study procedures and roles.

The PRecISion Medicine for Children with Cancer (PRISM) study is a national multicentre precision medicine clinical trial for children and adolescents with high-risk cancers in Australia (Australian and New Zealand Clinical Trials Registry (ANZCTR): NCT03336931). PRISM succeeds the Targeted Agents for high Risk Groups of paEdiatric Tumours (TARGET) pilot study [27]. Children and adolescents (≤ 21 years) are eligible for PRISM if they have a high-risk malignancy defined by an expected overall chance of survival of less than 30%, and an anticipated life expectancy ≥ six weeks. PRISM opened in September 2017 and aims to recruit up to 400 patients over a three-year period from eight paediatric oncology centres, with five years’ follow-up. PRISM aims to assess the feasibility and clinical utility of a new diagnostic service which includes tumour molecular profiling, in-vitro drug sensitivity testing and in-vivo drug modelling using patient-derived xenograft (PDX) models. Participation in PRISM may require an additional biopsy to provide appropriate tumour material. Actionable results are discussed at a biweekly MTB meeting, followed by reporting of clinical recommendations that are returned to families (Figure 1). 

#### 2.1.2. PRISM-Impact

We invited PRISM clinicians (including oncologists, pathologists, geneticists and genetic counsellors) and scientists (including bioinformaticians and laboratory scientists to participate in a mixed-methods prospective study (hereafter referred to as ‘PRISM-Impact’) to track their experiences of involvement in PRISM (Figure 1). Participants were eligible if they were investigators and/or had enrolled patient(s) in the PRISM study. PRISM-Impact interviews participants once at the start of PRISM, then annually. This paper presents data collected during the clinician and scientist interviews in the first year of PRISM (September 2017–September 2018). All participants provided their informed consent before they participated in the study. The study was conducted in accordance with the Declaration of Helsinki, and both PRISM and PRISM-Impact received Institutional Board Approval (17/02/15/4.06; HREC/17/HNE/29). 

### 2.2. Procedure

We invited clinicians and scientists to PRISM-Impact via a personalised email from the study co-ordinator (C.W.). Clinicians/scientists opted in/out by email. If the staff member did not respond to the invitation within two weeks, the study co-ordinator sent one reminder email. Any staff member who could not be reached after two failed attempts was deemed unreachable. Participants could choose to complete their interview over the telephone or in person, if they were based in Sydney, Australia. 

### 2.3. Data Collection

#### 2.3.1. Measures 

Demographics: Participants reported their age, number of years of professional practice, number of years working in paediatric oncology, and proportion of their professional time dedicated to research.

Knowledge of and confidence with genetics: To provide further context to oncologists’ perceptions of professional challenges, we asked seven items derived from previous research [28,29] to assess their perceived knowledge of genetics (rated as 1 = very good, 2 = good, 3 = poor, 4 = very poor). We also asked oncologists to rate their confidence in tasks related to somatic and germline testing (e.g., making treatment recommendations; 1 = very confident, 2 = moderately confident, 3 = a little confident, 4 = not confident at all). 

#### 2.3.2. Interview

A multidisciplinary team comprising oncology health professionals and psychosocial researchers developed the semi-structured interview schedule. Interview topics included: Hopes and expectations regarding precision medicine, early experiences with PRISM (including the MTB), and perceived professional challenges. Three researchers with postgraduate degrees in health sciences (J.V., P.T., M.W.; two females, one male) conducted the interviews which lasted 25 min on average (range 15–47 min). We audio-recorded the interviews which were then transcribed verbatim. 

### 2.4. Data Analysis

We analysed participants’ demographic and Likert scale data using descriptive statistics with the Statistical Package for the Social Sciences (SPSS version 25; IBM, Armonk, NY, USA). We used a thematic approach to analyse the qualitative interview data [30], with the support of coding software NVivo (QSR International Pty Ltd. Version 12, 2018). We created an initial deductive coding system based on the interview questions and coded the interviews line-by-line (B.M., J.V., E.D). We used matrix coding to compare the experiences of clinicians and scientists. B.M. developed the themes using an inductive coding approach, which was discussed with the study team until consensus was reached. 

## 3. Results

We invited 89 PRISM professionals to the study; of those, 62 opted in. Fifty-four went on to complete the interview (87% participation rate). Of the professionals interviewed, 18 clinicians (46%) and all scientists had been involved in TARGET, the PRISM pilot. The majority of clinicians (*n* = 31, 79%) were investigators on the PRISM study and most were oncologists (*n* = 35, 90%; Table 1). 

### 3.1. Knowledge of and Confidence with Genetics 

**Figure 2:**
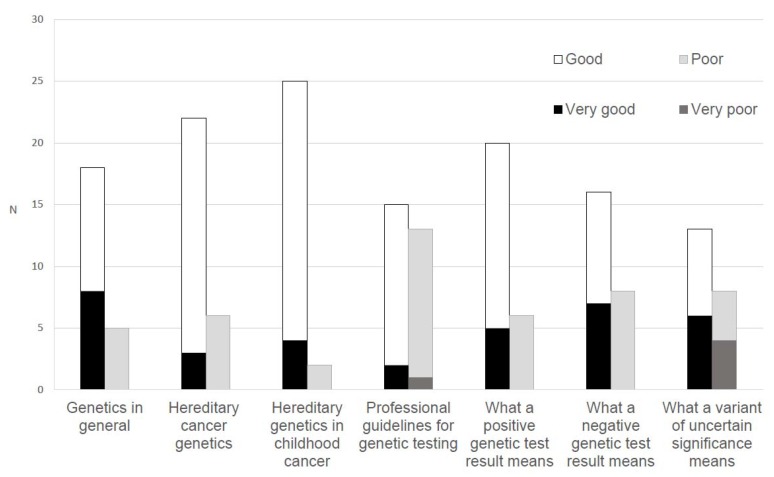
Oncologists’ (*n* = 35) ratings of their perceived knowledge of genetics.

Most oncologists indicated that their knowledge of the following domains was at least ‘good’: General genetics (*n* = 26, 83.9%), hereditary cancer genetics (*n* = 25, 80.7%), hereditary genetics in childhood cancer (*n* = 29, 93.5%), and the meaning of a positive test result (*n* = 25, 80.6%) and a negative test result (*n* = 23, 74.2%). Fewer rated ‘good’ to ‘very good’ knowledge of the meaning of a variant of uncertain significance (*n* = 19, 61.3%) and knowledge of professional guidelines for genetic testing (*n* = 17, 54.9%; Figure 2). 

**Figure 3:**
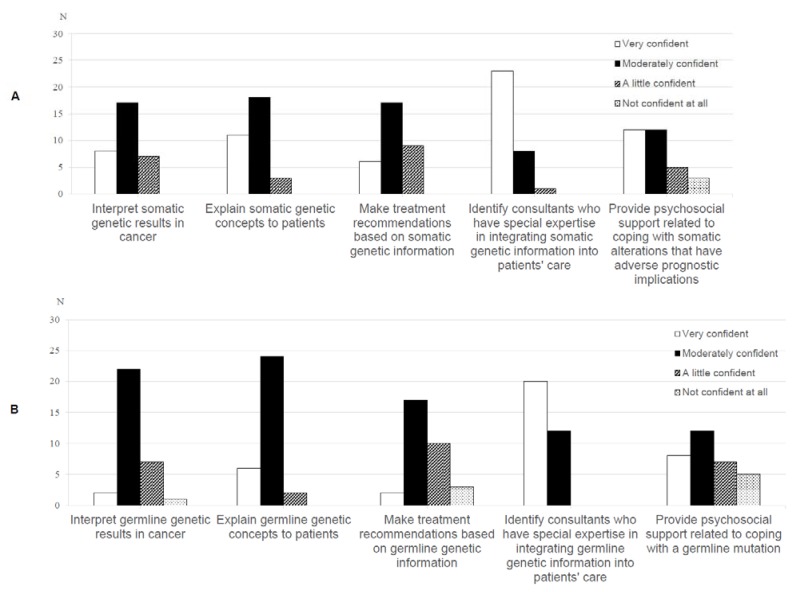
Oncologists’ (*n* = 35) ratings of their confidence with (**A**) somatic and (**B**) germline genetic test results.

A minority of oncologists were ‘very confident’ in interpreting (*n* = 8, 25%), explaining (*n* = 11, 34.4%) and making treatment recommendations (*n* = 6, 18.8%) based on somatic genetic test results. (Figure 3A). Thirteen oncologists (40.7%) were ‘not’ or ‘a little’ confident in making treatment recommendations based on germline genetic information (Figure 3B).

### 3.2. Interview

We identified eleven themes, some cross-cutting and some unique to the experiences of each participant group. We have organised the themes under two broad topic areas: early experiences with PRISM, and professional challenges (Table 2). If a small number of participants endorsed a specific profession (e.g. genetic counsellor), their quotes are characterised as ‘clinician-other’ or ‘scientist-other’ to preserve anonymity.

#### 3.2.1. Early Experiences with PRISM

Cautious optimism: Both clinicians and scientists expressed positive attitudes about their involvement in the PRISM trial and enthusiasm about its long-term potential to “help patients to live at least a slightly healthier, longer life” (ID 36, laboratory scientist). Clinicians expressed a more conservative viewpoint about the short-term outcomes of the trial:

*I’m not anticipating a whole lot of ‘miracle’ wins to be honest. I think there’ll be the occasional kid who appears to derive clinical benefit…but I think the majority of families will get closure out of it, rather than a miracle.*

(ID 49, oncologist)

Managing expectations: Both clinicians and scientists highlighted the importance of managing stakeholder expectations. There was a particular focus on managing the expectations of parents to avoid ‘false hope’, especially at the point of the child’s palliation:

*The difficulty is when you have a palliative patient and you’re still giving them access to a phase 1 study their interpretation is that ‘it is going to change things’.*

(ID 8, oncologist)

Some clinicians and scientists questioned whether the media and community excitement surrounding the trial was fuelling unrealistic expectations:

*PRISM has such a big buzz around it—you know, we had the Prime Minister come here…I think that they think PRISM is the ‘brand new personalised medicine program that will help save lives’, which is what we aim for, but we’re still setting it up.*

(ID 41, laboratory scientist)

A new way of working: Reflecting their stance of ‘cautious optimism’, clinicians commonly indicated that they thought precision medicine had the potential to change practices in paediatric oncology, albeit in the longer-term. As this oncologist reflected:

*I think it’ll be a little while before we see the bigger changes, but day-to-day I’m seeing the smaller changes. I’m seeing us being able to offer things to families we wouldn’t have otherwise.*

(ID 7, oncologist)

Most scientists indicated that their role in the PRISM trial was different to previously held positions, with its focus on clinically relevant outcomes. Some scientists anticipated a “change of culture” (ID 40, laboratory scientist) in their workplace as a shift occurred “…from a basic research lab into a diagnostic lab providing a service to the hospital…” (ID 40).

The MTB: a valuable forum, with room for improvement: Clinicians and scientists described their early experiences with the MTB as overwhelmingly positive. They perceived it as a valuable forum for building professional relationships and, for scientists, to gain insight into clinical decision-making processes. Clinicians valued the MTB for their own professional development, as explained here:

*The basic molecular biology of these things is still over my head. However, being able to learn from the clinician-scientists and all the laboratory staff who are running all the nitty-gritty of these tests really is informative for the clinician.*

(ID 7, oncologist)

Some clinicians and scientists highlighted a need for procedural improvements to the MTB, including systems to facilitate greater interdisciplinary communication and feedback about patient outcomes, to build an integrated knowledge base of “what works, and what does not work” (ID 47, scientist-other. As one clinician elaborated:

*What would be really useful would be to know what happens with a lot of the findings whether recommendations were enacted, or if any child benefited from it. I think that would be useful especially for future patients.*

(ID 1, clinician-other)

#### 3.2.2. Professional Challenges 

Participants described several anticipated or experienced challenges associated with working in a precision medicine trial.

Difficulty understanding and communicating results: Some oncologists experienced differences in sharing PRISM results with patients compared to other medical results. They indicated that the complexities associated with genomic data, and uncertainty surrounding treatment recommendations, often necessitated longer and more considered conversations with patients and/or their families:

*There is no knowing whether it will be effective. There is no evidence—not much evidence—you can give to back it up. There is just a lot more uncertainty around the discussion.*

(ID 7, oncologist)

Clinicians expressed that germline findings from the PRISM study had the potential to introduce an additional level of complexity to the care of patients and their families. Some clinicians had witnessed a negative psychological impact of germline information on the wider family at an already challenging time:

*The difficulty was that the parents found out when the child was very unwell and palliative…so they not only had to deal with that, but also the potential that [the mother] might also be at risk.*

(ID 1, clinician-other)

Some clinicians worried about the potential for the study to uncover germline variants with uncertain clinical impact for the child and wider family. Among clinicians without specialised genetics training, there was an expressed sense of “…that is not my area of expertise” (ID 19, oncologist).

Balancing risk of additional biopsies: Clinicians spoke of their “duty” (ID 4, oncologist) to help families balance the risk and benefits of additional biopsies required for PRISM. Some clinicians highlighted potential future cases which would contraindicate additional biopsies due to the potential for undue harm to the child:

*Say it was a brain tumour and the surgeon said “Well yes I can get a biopsy but it might cost vision”. Your starting premise has to be that the harm to the child is less than the potential benefits.*

(ID 20, oncologist)

Limited drug availability and access: Clinicians acknowledged uncertainty with regards to drug availability and access in the context of an identified therapeutic target. Some clinicians specifically mentioned drug cost (to the hospital and/or the family) as a prohibitive factor. As one oncologist explained:

*The big risk is that you find a therapeutic target and yes, there is a drug available, but we cannot get the drug…you build up hope and then take it away.*

(ID 3, oncologist)

Greater emotional impact with more awareness of the patient: Most scientists experienced a greater awareness of the patient and family impacts of their work than they had in previous roles, sometimes due to closer working relationships with the child’s clinical team and involvement in the MTB, which added “a certain gravity to the importance of what we’re doing” (ID 45, laboratory scientist). 

Some scientists described feeling an enhanced sense of responsibility and emotional impact, which was particularly related to the young age of the patient:

*If you see a kid who’s like two months old…you get moved by it quite a lot more than if you see the patient is 25 or 70 years old. That was the big psychological challenge.*

(ID 42, scientist-other)

Resisting urgency: Despite acknowledging new emotional challenges of their role, scientists also described the importance of pushing back against time pressures to enable them to “take the time to do it properly and be confident in the validity of the results” (ID 45, laboratory scientist). This scientist described their own experience of resisting this pressure:

*As the time gets closer and closer, there may be more hallway talk happening, like ‘what is happening with this patient’ and then things get missed. However, no, stick to the protocol, stick to the protocol.*

(ID 35, scientist-other)

Balancing translational outputs with academic productivity: Some scientists highlighted the professional tension associated with maintaining academic outputs in a translational research space and determining authorship within a large team of stakeholders:

*We need academic output to remain competitive in our careers. However, that is one of the challenges, when you invest so much in each patient. How do you translate that into academic outputs?*

(ID 43, scientist-other)

Logistical challenges of a national trial: Clinicians and scientists alike described inevitable procedural “kinks” (ID 34, laboratory scientist) associated with a new multicentre trial. They indicated that systems improvements, including better communication between sites, and smooth transfer of patient samples, largely hinged on the leadership and resources of the various sites:

*You need a champion at every site. It is a very smooth process within [site] but is that process going to be as smooth at the other centres? That takes resources and motivation.*

(ID 9, oncologist)

## 4. Discussion

To our knowledge, our study is the first to assess clinicians’ and scientists’ experiences and professional challenges in the early stages of a national precision medicine trial for high-risk childhood cancers. In line with research reporting healthcare professional optimism about precision medicine [11], both clinicians and scientists in our study described early positive attitudes towards the PRISM trial. At the same time, participants were conservative in predicting changes to outcomes for current patients, and placed focus on the importance of managing families’ expectations to avoid engendering ‘false’ hope. Clinicians’ expressed concerns about drug availability and cost further highlights that the successful delivery of paediatric precision medicine hinges on easy access to approved agents and/or experimental agents via clinical trials [1,2]. Some overlapping professional challenges for clinicians and scientists were evident, including logistical challenges associated with a large multicentre trial. Both clinicians and scientists valued involvement in the MTB, supporting research that MTBs are not only beneficial for patients but provide a forum for continued medical education [31,32]. As has been described elsewhere, our participants also acknowledged the challenges associated with building a clinically useful evidence base to inform future treatment decisions [32,33], which is a long-term goal of PRISM. 

Echoing other research [12,13,34,35,36], clinicians in our study reported challenges in understanding and communicating genomic data. The successful mainstreaming of precision medicine genomics in paediatric cancer care will require further education of the current workforce and ultimately the development of a new workforce, specifically trained to manage the challenges of this model which is emerging as the new gold standard of care [10,37,38]. Our finding that the minority of oncologists were highly confident in interpreting, explaining and making treatment recommendations based on somatic genetic test results and germline cancer predisposition speaks to the acknowledged need for education and training in these areas [35,39] and is critical information for medical educators and accreditation boards. Our data supports the need for greater integration of oncology and hereditary cancer clinical genetics services to meet families’ needs, and to support non-genetics-trained clinicians, as germline genomic information is increasingly available [37]. Enhancing non-genetics trained clinicians’ knowledge of germline cancer predisposition, thereby increasing confidence in anticipating and managing potential family impacts, may go some way in reducing the unsustainable burden on hereditary cancer clinics as precision medicine advances. 

Our study also revealed that PRISM scientists were experiencing a dynamic process of navigating new ways of working. Key tensions included the emotional demands and time pressures of working with ‘real’ patient data, balanced against a desire to maintain rigorous adherence to protocols. Scientists in our study also described difficulties balancing academic productivity with translational outputs. Our data indicates that protocols for providing support to scientists, including debriefing procedures, may be warranted to avoid deleterious occupational stress. Furthermore, if service reorganisation is to take place for scientists in the context of precision medicine, expectations regarding academic outputs and authorship need to be defined concurrently. 

The key strength of our novel study is in the inclusion of multiple stakeholders recruited nationally. We had a very high participation rate and were the first study to closely examine the perspectives of scientists. The study would have been strengthened by the inclusion of an even wider variety of perspectives from other clinicians involved in the PRISM trial, for example, surgeons and allied health. Although we used a best-practice reflexive approach (e.g., multiple interviewers) we acknowledge that qualitative research is characterised by an inherent risk of researcher bias. As half of the clinicians and all scientists in our study were involved in the PRISM pilot, and the majority of clinicians were PRISM investigators, our data may be biased by a particular enthusiasm for, and experience with, precision medicine. The professional challenges identified in this study are likely to be magnified in the wider workforce. We also had a higher response from professionals at the PRISM main site, precluding us from more closely examining the experiences of staff at other sites. PRISM-Impact offers opportunities to purposely target these groups for participation as the trial progresses. 

## 5. Conclusions

Clinicians and scientists are optimistic about the potentials of precision medicine but acknowledged several professional challenges. Closer examination of these challenges as they evolve over the course of the trial, while integrating the perspectives of a wider range of stakeholders, will drive the ongoing development of a workforce equipped to manage the tasks of paediatric precision medicine. 

## Figures and Tables

**Table 1:** Participant characteristics (*n* = 54).

	Clinicians (*n* = 39)	Scientists (*n* = 15)
Profession, clinicians, n (%) Oncologist Pathologist Geneticist Genetic counsellor	35 (90) 2 (4) 1 (3) 1 (3)	
Profession, scientists, n (%) Laboratory scientist ^1^ Bioinformatician Technical support		11 (73) 3 (20) 1 (7)
Site, n (%) PRISM main site Others	11 (28) 28 (72)	11 (73) 4 (27)
PRISM investigators ^2^, n (%)	31 (79)	1 (7)
PRISM pilot ^3^, n (%)	18 (46)	15 (100)
Age, mean (SD), range, y	47 (9), 30–73	36 (9), 25–57
Sex, no. (%) Male Female	18 (46) 21 (54)	9 (60) 6 (40)
No. years professional practice, mean (SD), range	20 (12), 5–45	7 (5), 1-20
No. years paediatric oncology practice, mean (SD), range	16 (10), 2–43	5 (5), 1–20
Time dedicated to research (%), mean (SD), range	25 (19), 0–80	87 (28), 10–100

^1^ Scientists conducting laboratory experimentation and curation scientists. ^2^ Including principal and associate investigators and study committee members. ^3^ Involvement in the PRISM pilot study, ‘TARGET’.

**Table 2:** Summary of themes from the clinician and scientist interviews.

Topic	Themes
Early experiences with PRISM	*Common to both groups* • Cautious optimism • Managing expectations • A new way of working • The MTB: a valuable forum, with room for improvement
Professional challenges	*Clinicians* • Difficulty understanding and communicating results • Balancing risk of additional biopsies • Limited drug availability and access *Scientists* • Greater emotional impact with more awareness of the patient • Resisting urgency • Balancing translational outputs with academic productivity *Common to both groups* • Logistical challenges of a national trial
